# Risk factors associated with multiple organ damage in childhood**-**onset systemic lupus erythematosus

**DOI:** 10.3389/fped.2023.1301201

**Published:** 2023-11-28

**Authors:** Thanaporn Puengpipattrakul, Butsabong Lerkvaleekul, Kwanchai Pirojsakul, Soamarat Vilaiyuk

**Affiliations:** ^1^ Department of Pediatrics, Faculty of Medicine Ramathibodi Hospital, Mahidol University, Bangkok, Thailand; ^2^ Division of Rheumatology, Department of Pediatrics, Faculty of Medicine Ramathibodi Hospital, Mahidol University, Bangkok, Thailand; ^3^ Division of Nephrology, Department of Pediatrics, Faculty of Medicine Ramathibodi Hospital, Mahidol University, Bangkok, Thailand

**Keywords:** disease damage, connective tissue disease, pediatric, infection, disease activity, ocular, predictors, SLE

## Abstract

**Objective:**

Previous studies have shown that approximately 39%–65% of patients with childhood-onset systemic lupus erythematosus (cSLE) have damage in at least one organ. Data on risk factors for organ damage in cSLE remain limited, especially in Asian populations. This study was conducted to evaluate the incidence of cSLE and identify the risk factors for accumulated organ damage in patients with cSLE.

**Methods:**

This was a retrospective study. Patients aged <18 years who were diagnosed with cSLE between 2008 and 2020 were enrolled. Information on baseline characteristics, treatment, and disease activity assessed using the Systemic Lupus Erythematosus Disease Activity Index 2000 (SLEDAI-2K) was collected from diagnosis until the most recent visits were reviewed from medical records. Disease damage was measured using the Systemic Lupus International Collaborating Clinics/American College of Rheumatology Damage Index (SDI).

**Results:**

A total of 134 patients with a mean age at diagnosis of 11.2 ± 2.9 years were enrolled. The median duration of treatment was 4.7 (interquartile range 2.8–7.1) years. Forty patients (29.9%) had irreversible organ damage (SDI > 1) with an incidence rate of 5.7 events per 100 person-years. The most frequent type of organ damage was ocular (11.1%), followed by musculoskeletal (8.9%) and neurological (7.4%). High disease activity at diagnosis (SLEDAI-2K ≥ 12) (odds ratio [OR] 3.19, 95% confidence interval [CI] 1.32–7.68), infection (OR 3.73, 95% CI 1.60–8.67), and mycophenolate mofetil use (OR 3.62, 95% CI 1.45–9.03) were predictors of organ damage. The median time to disease damage in patients with SLEDAI-2K scores ≥12 at diagnosis was 6.5 years (95% CI 5.77–7.36; *P* = 0.004).

**Conclusion:**

Physicians should be aware of organ damage in patients with cSLE, particularly those with high disease activity at initial presentation, those who are receiving mycophenolate mofetil therapy, and those with an infection.

## 1. Introduction

Systemic lupus erythematosus (SLE) is a multisystem autoimmune disease with variable clinical presentations. Childhood-onset SLE (cSLE) is defined as the onset of SLE before the age of 18 years. The median age of onset is 12 years. cSLE accounts for 10%–20% percent of SLE cases, and the overall incidence of SLE varies between 0.36 and 2.5 per 100,000 population (1). Although cSLE is less common than adult-onset SLE, it is a severe form of the disease with a higher rate of organ involvement. Organ damage accrual in patients with SLE persists despite the decades-long improvement in the survival rate driven by the introduction of novel SLE medications. Thus, physicians should consider not only the treatment outcomes in patients with SLE but also the morbidity and quality of life of these patients.

In addition to SLE disease, several factors, including the environment, disease activity, drug side effects, and comorbid conditions, can cause long-term organ damage. The Systemic Lupus International Collaborating Clinics/American College of Rheumatology Damage Index (SDI) is a useful tool that helps monitor the progression over time of organ damage from the disease or its complications. The SDI detects permanent organ damage at least 6 months after disease onset and consists of 41 items in 12 organs or systems (score range: 0–47), including the ocular, neuropsychiatric, renal, pulmonary, cardiovascular, peripheral vascular, gastrointestinal, musculoskeletal systems; skin; premature gonadal failure; diabetes; and malignancy (2).

The literature supports that approximately 39%–65% of patients with SLE have damage to at least one organ (SDI ≥ 1), and SDI scores range between 0.6 and 2.3 (3–7). According to previous studies, the most frequent types of organ damage were ocular, musculoskeletal, neuropsychiatric, and renal (8). Three multicenter survey studies in 39 countries reported that approximately 40% of patients with cSLE had disease damage to at least one organ (4). Consistent with findings from another study, the most damaged organs were the kidneys (9). Canadian studies showed that risk factors included ethnicity, disease activity, a laboratory at diagnosis, and medications (10, 11). Major organ involvement was also a factor for irreversible damage (11–13). The SDI score increased over time in an Asian study (14).

Asian data on cSLE frequency and the predictors of disease damage remain limited. Ethnicity or socioeconomic issues in a setting of limited resources can influence SLE severity and organ damage accrual. Therefore, we aimed to determine the incidence of irreversible organ damage and the risk factors that affect accumulated damage in cSLE.

## 2. Materials and methods

This was a retrospective cohort study. We recruited patients with SLE who had regular follow-ups for at least 1 year between 2008 and 2020 at the Faculty of Medicine Ramathibodi Hospital and were diagnosed with SLE in accordance with either the American College of Rheumatology (ACR) 1997 criteria (15) or Systemic Lupus International Collaborating Clinics (SLICC) 2012 criteria (16) before the age of 18 years. Patients with SLE who had underlying diseases that affected irreversible organ damage were excluded. Additionally, patients who lacked complete information for critical factors or those who were lost to follow-up were excluded from the study. Patient data were reviewed from medical records. Ethical approval was obtained from the ethics committee of the Faculty of Medicine Ramathibodi Hospital, Mahidol University, and the informed consent requirement was waived given the retrospective study design.

### 2.1. Data collection

We collected demographic data, including age, sex, weight, body mass index, other underlying diseases, duration of illness, duration of treatment, clinical manifestations at diagnosis, and laboratory data, including complete blood count, erythrocyte sedimentation rate, complement levels, antinuclear antibody (ANA), anti-double stranded DNA (anti-dsDNA), anti-phospholipid antibodies, direct Coombs' test, serum creatinine, urinalysis, and urine protein creatinine ratio. We further reviewed medications with which patients had been treated in all visits, including the cumulative dose of corticosteroid, the cumulative dose of cyclophosphamide, hydroxychloroquine, azathioprine, methotrexate, and mycophenolate mofetil (MMF). Information on infections during the disease was also collected.

The Systemic Lupus Erythematosus Disease Activity Index 2000 (SLEDAI-2K) (17) was used to assess disease activity at diagnosis and every 3–6 months until the most recent visit. The cumulative SLEDAI-2K score was calculated by summing the scores serially from the date of diagnosis to the most recent visit using the trapezoidal rule (18). Additionally, the severity of the disease flare was determined by (1) a >12-point change in the SLEDAI-2K score; (2) worsening of disease in involved organs or the involvement of new organs, resulting in neuropsychiatric lupus erythematosus, vasculitis, nephritis, myositis, platelet <60,000/mm^3^, or hemolytic anemia with hemoglobin <7 mg/dl that required the doubling or the use of prednisolone >0.5 mg/kg/day; (3) hospitalization for active SLE; (4) prednisolone >0.5 mg/kg/day; (5) the addition of new immunosuppressive medications; or (6) physician global assessment >2.5, according to the Safety of Estrogen in Lupus Erythematosus National Assessment trial definition of severe flares (19). The remission on medication was defined by clinical SLEDAI-2K, which excluded anti-dsDNA and complement levels, equal to zero for one year, along with physician global assessment of disease activity <0.5 (0–3 visual analogue scale), daily prednisolone dose ≤5 mg/day or ≤0.2 mg/kg/day for patients whose body weight <25 kg, with or without hydroxychloroquine and maintenance immunosuppressive medications (20).

### 2.2. Disease damage

We used the SDI score (2) to measure accumulated damage, which was defined as a change in an organ that is irreversible for at least 6 months. The SDI score was assessed at the most recent visit, in which the time to disease damage was determined with a review of medical records. We classified patients into two groups—patients with and without organ damage—according to the SDI score (SDI score cut-off ≥1). In this study, our patients had regular ophthalmic examinations every 6–12 months.

### 2.3. Statistical analysis

Descriptive data were reported as mean and standard deviation (SD) or median and interquartile range (IQR) for continuous variables and the frequency as a percentage for categorical variables. We clarified the remaining number of variables in the manuscript if data were missing. Parameters of patients with and without disease damage were compared using Student's *t*-test or the Mann–Whitney *U* test for continuous data and the Chi-squared or Fisher's exact tests for categorical data. Logistic regression analysis was performed to determine the predictors of organ damage in patients with cSLE and presented as an odds ratio (OR). The Kaplan–Meier survival and Cox regression analyses were performed to determine the predictors of organ damage related to time. A mixed-effect linear regression analysis was used to evaluate the differences in variables between groups over time. Statistical analysis was performed using SPSS version 22 (IBM Corp., Armonk, NY, USA). Significance levels were set at *P* < 0.05.

## 3. Results

A total of 159 patients were included in this study; 25 patients were excluded (9 were transferred to other hospitals, and 16 were irregularly followed or lost to follow-up) (Supplementary Figure S1). Ultimately, we enrolled 134 patients, of whom 109 (81%) were women and 25 (19%) were men. The mean age at diagnosis was 11.2 ± 2.9 years and the median duration of treatment was 4.7 (IQR 2.8–7.1) years. The most frequent system involvement was hematologic (75%), followed by mucocutaneous (61%), musculoskeletal (38%), and renal (lupus nephritis, class III–V; 34%). Almost all of the patients received corticosteroids (99%) and hydroxychloroquine (95%) as treatment. Other medications were azathioprine (77%), cyclophosphamide (47%), and MMF (24%). The baseline disease activity according to the SLEDAI-2K score was 12.6 ± 6.9 (Table 1).

**Table 1:** Baseline characteristics of cSLE patients with and without disease damage.

	All patients	No disease damage	Disease damage	*P* value
	*N* = 134	*n* = 94	*n* = 40	
Patient characteristics				
Male/female, *n* (%)	25/109 (18.6/81.4)	17/77 (18.1/81.9)	8/32 (20.0/80.0)	0.795
Age at diagnosis (y)	11.2 ± 2.9	11.4 ± 3.0	10.7 ± 2.5	0.198
Duration of disease (y)^a^	5.1 (3.6, 7.7)	4.6 (2.9, 6.5)	6.5 (4.5, 8.5)	0.022*
Duration of treatment (y)^a^	4.7 (2.8, 7.1)	4.4 (2.7, 6.3)	5.9 (4.2, 8.0)	0.034*
Duration prior to diagnosis (m)^a^	1.2 (0.5, 3.1)	1.3 (0.4, 2.7)	1.0 (0.5, 7.2)	0.894
BMI (kg/m^2^)	23.1 ± 5.4	22.9 ± 5.2	23.3 ± 5.8	0.710
Clinical manifestations at diagnosis				
SLEDAI-2K score	12.6 ± 6.9	11.6 ± 6.5	14.9 ± 7.4	0.009*
Fever, *n* (%)	27 (20.1)	21 (22.3)	6 (15.0)	0.332
Hematological, *n* (%)	100 (74.6)	68 (72.3)	32 (80.0)	0.351
Neurological, *n* (%)	18 (13.4)	8 (8.5)	10 (25.0)	0.010*
Mucocutaneous, *n* (%)	82 (61.2)	56 (59.6)	26 (65.0)	0.555
Serosal, *n* (%)	24 (17.9)	11 (11.7)	13 (32.5)	0.004*
Musculoskeletal, *n* (%)	51 (38.1)	39 (41.5)	12 (30.0)	0.210
Lupus nephritis class III-V, *n* (%)	46 (34.3)	31 (33.0)	15 (37.5)	0.614
Vasculitis, *n* (%)	26 (19.4)	14 (14.9)	12 (30.0)	0.043*
Gastrointestinal, *n* (%)	7 (5.2)	6 (6.4)	1 (2.5)	0.674
Cumulative SLEDAI-2K^a^	2,705.5 (1,263.5, 5,193.9)	2,435.0 (1,194.0, 4,403.1)	3,592.0 (1,539.8, 7,610.8)	0.066
Laboratory				
Hematocrit	30.8 ± 7.1	30.7 ± 7.3	31.3 ± 6.6	0.662
White blood cell (×10^3 ^cells/mm^3^)^a^	5.0 (3.5, 8.3)	5.4 (4.1, 8.3)	4.2 (3.1, 8.6)	0.165
Absolute lymphocyte count (×10^3 ^cells/mm^3^)^a^	1.6 (1.1, 2.4)	1.8 (1.1, 2.4)	1.3 (1.1, 2.4)	0.424
Leukopenia/lymphopenia, *n* (%)	60 (49.2)	37 (43.5)	23 (62.2)	0.058
Platelet (×10^3 ^cells/mm^3^)^a^	232.0 (140.0, 319.0)	234.0 (150.0, 327.5)	215.0 (114.0, 312.0)	0.466
Thrombocytopenia, *n* (%)	30 (24.0)	19 (21.6)	11 (29.7)	0.331
Anti-dsDNA, *n* (%)	98 (77.8)	68 (77.3)	30 (78.9)	0.836
C3 levels (0.9–1.8 g/L)^a^	0.56 (0.29, 0.87)	0.61 (0.29, 0.94)	0.45 (0.28, 0.77)	0.157
C4 levels (0.1–0.4 g/L)^a^	0.07 (0.03, 0.15)	0.07 (0.03, 0.15)	0.06 (0.04, 0.13)	0.756
Hypocomplementemia, *n* (%)	97 (78.9)	65 (76.5)	32 (84.2)	0.331
ESR (mm/h)^a^	58.0 (26.0, 87.0)	57.0 (26.5, 88.0)	62.5 (21.0, 82.5)	0.285
DCT, *n* (%)^b^	58 (59.2)	42 (60.9)	16 (55.2)	0.600
aPL, *n* (%)^c^	12 (11.4)	8 (11.0)	4 (12.5)	1.000
Treatment				
Prednisolone, *n* (%)	133 (99.3)	93 (98.9)	40 (100.0)	1.000
Cumulative corticosteroid dose (mg/kg)^a^	347.29 (218.2, 551.1)	318.9 (184.2, 497.5)	486.2 (243.2, 744.2)	0.004*
Cyclophosphamide, *n* (%)	63 (47.0)	36 (38.3)	27 (67.5)	0.002*
Cumulative cyclophosphamide (mg/m^2^)	2,630.6 ± 3,261.7	2,269.7 ± 3,238.3	3,478.8 ± 3,197.3	0.049*
Mycophenolate mofetil, *n* (%)	32 (23.9)	15 (16.0)	17 (42.5)	0.001*
Azathioprine, *n* (%)	103 (76.9)	73 (77.7)	30 (75.0)	0.738
Hydroxychloroquine, *n* (%)	127 (94.8)	89 (94.7)	38 (95.0)	1.000
Methotrexate, *n* (%)	6 (4.5)	3 (3.2)	3 (7.5)	0.363
Cyclosporine, *n* (%)	3 (2.2)	2 (2.1)	1 (2.5)	1.000
Immunoglobulin, *n* (%)	6 (4.5)	3 (3.2)	3 (7.5)	0.363
Rituximab, *n* (%)	2 (1.5)	0 (0.0)	2 (5.0)	0.088
ACEI, *n* (%)	52 (38.8)	30 (31.9)	22 (55.0)	0.012
Tacrolimus, *n* (%)	4 (3.0)	0 (0.0)	4 (10.0)	0.007*
Hemodialysis, *n* (%)	5 (3.7)	0 (0.0)	5 (12.5)	0.002*
TNF inhibitor, *n* (%)	1 (0.7)	0 (0.0)	1 (2.5)	0.299

BMI, body mass index; SLEDAI-2K, Systemic Lupus Erythematosus Disease Activity Index 2000; C, complement; ESR, erythrocyte sedimentation rate; DCT, direct Coombs' test; aPL, antiphospholipid antibodies; ACEI, angiotensin-converting enzyme inhibitor; TNF inhibitor, tumor necrosis factor inhibitor.

* *P* < 0.05 was set as significance.

^a^ Median (IQR).

^b^ Total number = 98 (69 in no disease damage, 29 in disease damage).

^c^ Total number = 105 (73 in no disease damage, 32 in disease damage).

### 3.1. Disease damage

**Figure 1:**
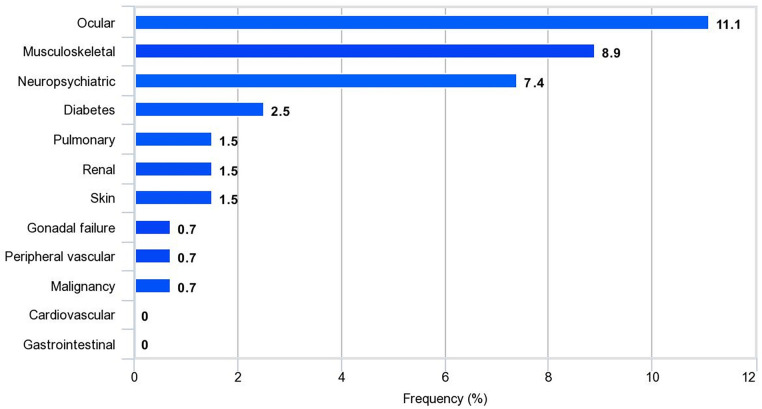
Frequency of organ damage in SLE patients (*n* = 134).

Forty patients (30%) had disease damage. The median SDI score was 1 (IQR 1–2) and the maximum SDI score was 4. The incidence of disease damage was 5.65 events per 100 person-years. The most frequent types of organ damage were ocular (11%), followed by musculoskeletal (9%) and neuropsychiatric (7%; Figure 1). Cataracts, avascular necrosis, and osteoporosis with fractures were the most serious diseases in this study; none of the patients had damage to the cardiovascular and gastrointestinal systems (Supplementary Table S1).

**Figure 2:**
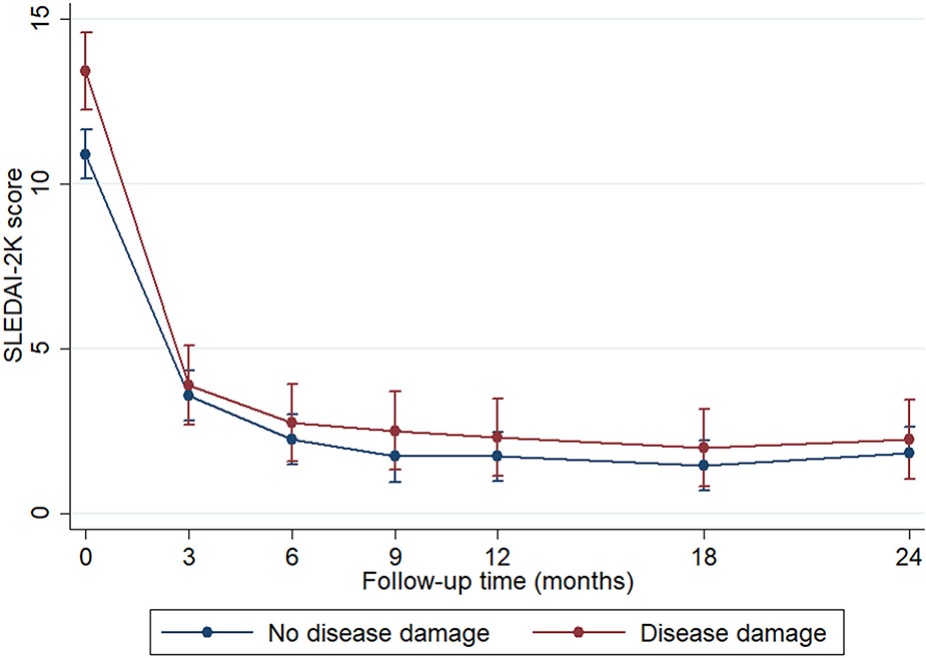
Disease activity (SLEDAI-2K score) during the 24 months of follow-up. Data represented the mean and standard error and were analyzed by mixed-effect regression analysis. *P < 0.05 was set as significance. The blue line represents patients without disease damage (*N* = 94), and the red line represents patients with disease damage (*N* = 40).

The mean age at disease damage was 13.5 ± 2.9 years, and the mean time to disease damage was 8.23 years [95% confidence interval (CI) 7.28–9.18] after diagnosis. We observed that sex, age at diagnosis, and duration of prior treatment were not significantly different between patients with organ damage and those without organ damage. However, patients with organ damage had a longer duration of treatment compared with patients without organ damage (5.9 [IQR 4.2–8.0] vs. 4.4 [IQR 2.7–6.3] years, *P* = 0.034). Moreover, patients with organ damage had higher disease severity at diagnosis than those without organ damage (mean SLEDAI-2K 14.9 ± 7.4 vs. 11.6 ± 6.5, *P* = 0.009). During the disease course, SLEDAI-2K scores decreased at the same rate in both groups after the patients received treatment (Figure 2). Cumulative SLEDAI-2K scores during the disease course were not significantly different between the groups (3,592.0 [IQR 1,539.8–7,610.8] vs. 2,435.0 [IQR 1,194.0–4,403.1], *P* = 0.066). Higher frequencies of neurological involvement, serositis, and vasculitis were also observed in patients with organ damage. The laboratory results were not statistically different between the groups, including hematocrit, absolute lymphocyte count, platelet count, erythrocyte sedimentation rate, direct Coombs' test, anti-dsDNA, and complement levels. In addition, the median time to remission on medication between patients without organ damage (1.70 years, 95% CI 1.15–2.24) and patients with organ damage (1.78 years, 95% CI 1.53–2.03), *P* = 0.734) were not different. However, the cumulative corticosteroid dose, the cumulative cyclophosphamide dose, and MMF use were significantly higher in patients with organ damage than those without organ damage (Table 1). Additionally, the duration of corticosteroid use was 1.5 (IQR 0.5–3.5) years before detecting organ damage.

### 3.2. Predictors of disease damage

**Figure 3:**
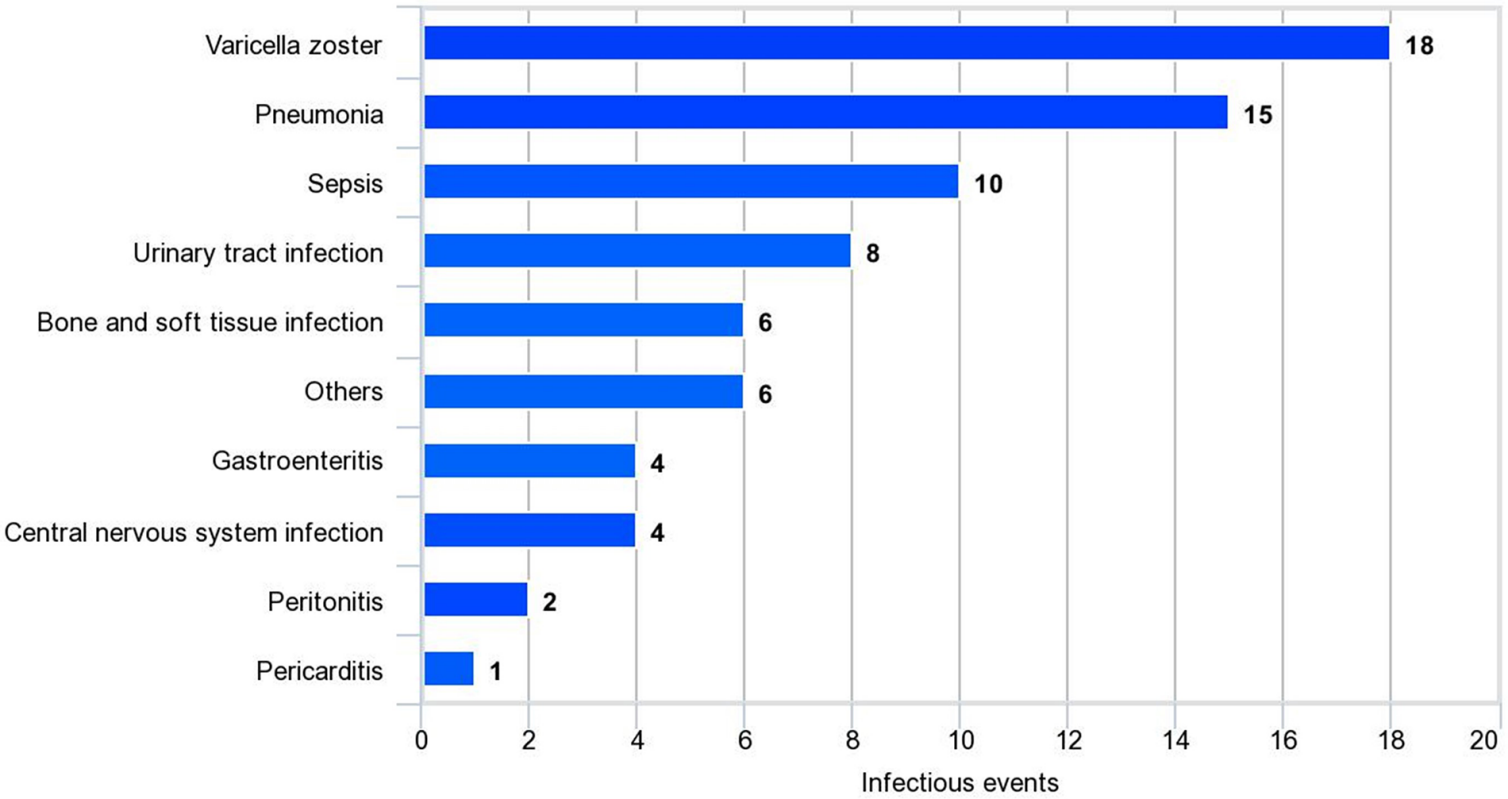
Infectious events in SLE patients. Other infections include Coronavirus, dengue virus, influenza virus, cytomegalovirus, and non-tuberculous mycobacteria.

Risk factors for disease damage were analyzed using logistic regression analysis. In the univariate analysis, we observed significant factors, including duration of treatment ≥5 years, serositis, neuropsychiatric lupus (NPSLE), SLEDAI-2K ≥ 12 at diagnosis, cumulative steroid dose, cumulative cyclophosphamide dose, MMF, severe flare, and infection. The multivariate analysis indicated that the associated factors were SLEDAI-2K ≥ 12 at diagnosis (OR 3.19, 95% CI 1.32–7.68, *P* = 0.010), infection (OR 3.73, 95% CI 1.60–8.67, *P* = 0.002), and MMF use (OR 3.62, 95% CI 1.45–9.03, *P* = 0.006; Table 2). Varicella zoster virus infection was the most common infection in this study, followed by pneumonia and sepsis (Figure 3). Additional information about infectious agents is shown in Supplementary Table S2.

**Table 2:** Predictors of disease damage in childhood-onset SLE patients.

Factors	Univariate analysis		Multivariate analysis	
	OR (95% CI)	*P* value	OR (95% CI)	*P* value
SLEDAI-2K ≥ 12 at diagnosis	3.00 (1.34–6.69)	0.007*	3.19 (1.32–7.68)	0.010*
MMF	3.89 (1.69–8.98)	0.001*	3.62 (1.45–9.03)	0.006*
Infection	4.25 (1.03–9.36)	<0.001*	3.73 (1.60–8.67)	0.002*
Serositis	3.63 (1.46–9.05)	0.006*		
NPSLE	3.58 (1.29–9.92)	0.014*		
Duration of treatment ≥5 years	2.57 (1.18–5.59)	0.017*		
Cumulative corticosteroid ≥560 mg/kg	3.25 (1.42–7.46)	0.005*		
Cumulative IVCY ≥3,500 mg/m^2^	2.26 (1.06–4.79)	0.034*		
Severe flare	2.86 (1.12–7.30)	0.028*		

* *P* < 0.05 was set as significance.

SLEDAI-2K, Systemic Lupus Erythematosus Disease Activity Index 2000; NPSLE, Neuropsychiatric lupus; MMF, Mycophenolate mofetil; IVCY, intravenous cyclophosphamide.

**Figure 4:**
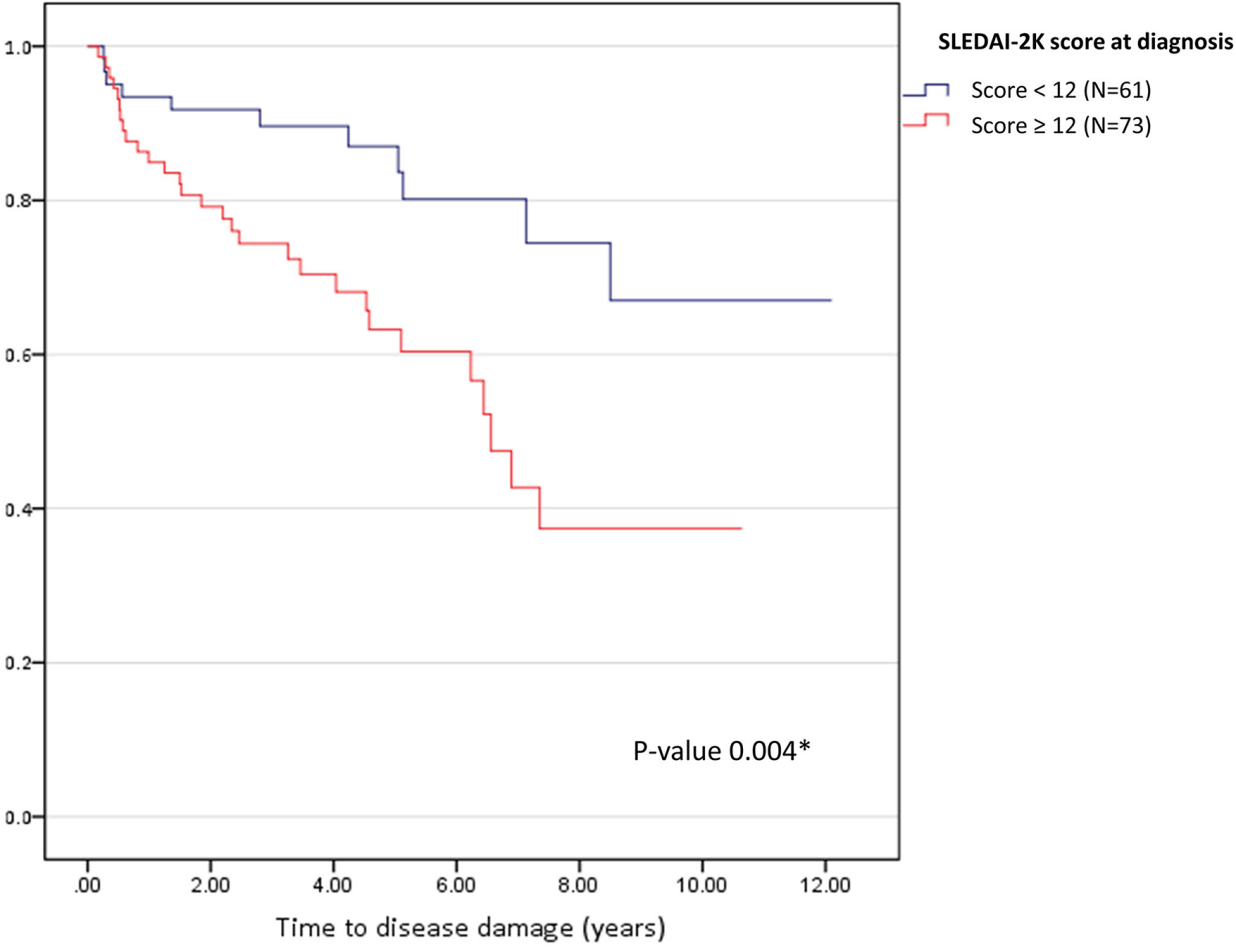
Kaplan-Meier curve for time to disease damage according to disease activity at diagnosis. The blue line represents SLEDAI-2K < 12 (*N* = 61), and the red line represents SLEDAI-2K ≥ 12 (*N* = 73). *P < 0.05 was set as significance.

The Kaplan–Meier curve and Cox regression analysis indicated that SLEDAI-2K ≥ 12 at diagnosis, which represents high disease activity, was the only significant predictor (hazard ratio 2.70, 95% CI 1.35–5.43, *P* = 0.005) of disease damage. The median time to disease damage in patients with SLEDAI-2K ≥ 12 at diagnosis was 6.5 years (95% CI 5.77–7.36; *P* = 0.004; Figure 4**)**.

## 4. Discussion

We revealed that one-third of the patients with cSLE in our study had disease damage after a median follow-up period of 5 years. The most prevalent type of irreversible organ damage observed was cataracts, followed by avascular necrosis, osteoporotic fracture, and cognitive impairment or major psychosis; corticosteroid usage contributed to some of these conditions. These findings are consistent with those in the literature, which has also reported musculoskeletal, ocular, renal, central nervous system, and skin damage as common types of organ impairment (6, 13, 21–25). Beyond providing valuable insights into disease damage patterns, we identified predictors of disease damage. In the univariate analysis, several factors, including severe flares, cumulative corticosteroid dose, cumulative cyclophosphamide dose, longer disease duration, NPSLE, and serositis, were associated with disease damage. However, the multivariate analysis indicated that only three factors were predictors of disease damage: a high SLEDAI-2K score at the time of diagnosis, MMF use, and infection. In patients with a high SLEDAI-2K score (≥12) at disease diagnosis, the median time to disease damage was approximately 6.5 years. Our study emphasizes the importance of early disease activity assessment and careful management in mitigating long-term organ damage risk. Identifying the predictors of disease damage can allow better tailoring of treatment approaches and improvement in patient outcomes.

The frequency of organ damage varied between studies because of several factors, including participant age, ethnicity, disease activity, organ involvement, and treatment regimen. Previous studies have reported that non-Caucasian patients with SLE have higher disease activity compared with Caucasians (26–32). Renal damage was most frequently observed in African American and Hispanic patients (33, 34), and NPSLE was more common in African Americans than in Caucasians (3, 35). Although previous studies have reported an increased rate of disease damage in African American patients with SLE with renal and/or CNS involvement (36), a Canadian study did not find significant differences in disease damage across ethnic groups (3). Although the literature reports conflicting findings about ethnicity, a previous study revealed that the prevalence of organ damage in Asian patients was similar to that in Caucasians (37). Older age at diagnosis is another factor associated with organ damage in SLE, as indicated by several studies (38–40). Moreover, patients with late-onset SLE had higher damage scores and exhibited different damage patterns (41). Additionally, socioeconomic conditions, including poverty and limited access to healthcare, were highlighted as significant factors in the LUMINA study and were more important than genetic factors (42).

In our study, the frequency of disease damage in cSLE was comparable to that in a Hong Kong study (13). The genetic similarity of the participants in the Hong Kong study and the patients in our investigation can be attributed to the Chinese origin of most Thai people. Furthermore, patients in both studies shared similar clinical characteristics. However, a study in Mexico reported a higher frequency of disease damage compared with that in our study. This difference can be explained by the inclusion of a larger number of patients with NPSLE in the Mexican study, in which neurological involvement emerged as a predictive factor for disease damage (12). Similarly, the 2002 study by Brunner et al. (10) found a higher frequency of disease damage compared with that in our study. This discrepancy may be explained by the higher proportion of patients with lupus nephritis included in the 2002 study compared with that in our study. Moreover, Brunner et al. enrolled patients from 1990 to 1998, whereas our research was conducted in a different period. The difference in the study start dates may be significant given that treatment regimens and patient care may have improved over time. Notably, the pre-2000 study start was a significant risk factor for SDI progression in 5 years (43).

In our study, we noticed a higher number of patients with SLE with neurological involvement, serositis, and vasculitis among those who experienced disease damage compared with patients who did not have disease damage. However, none of these factors were predictors of disease damage. Although previous reports have shown increased SDI scores in patients with serositis and vasculitis, none of these studies have revealed a significant association with disease damage (44–46). Nonetheless, these symptoms may have contributed to a more severe form of SLE and should be noted. In our study, although neurological involvement was a significant predictor of organ damage in the univariate analysis, the association did not persist in the multivariate analysis. This result may be attributable to the stronger association of disease damage with a high SLEDAI-2K score at the time of diagnosis, which already represents more severe disease in major organs, including neurological involvement in NPSLE. Because renal involvement is more common in cSLE than in adult-onset SLE (21, 23), cumulative steroid use in these patients causes more ocular damage (particularly cataracts) and avascular necrosis in children than in adults (23). Furthermore, our study showed that patients who received MMF had an additional risk of disease damage, which may indicate a more severe and refractory form of the disease in these patients.

Another important finding of our study was the association between disease damage and infection, which aggravated disease flares in patients with SLE. Conversely, a previous study reported that disease flares were a predictor of infection-related mortality in patients with SLE (47). Infections are commonly observed in patients with SLE and remain the cause of morbidity and mortality (48). The increased risk of infection in patients with SLE who receive immunosuppressive medications is well known. Moreover, impaired immune regulation in innate and adaptive immunity in patients with SLE further contributes to susceptibility to infections (49). Previous evidence has shown that some pathogens are involved in the pathogenesis of SLE. One commonly proposed mechanism is the molecular mimicry of infectious agents, which leads to autoreactive B cell and T cell activation (50, 51). Another mechanism is bystander activation, in which the initial immune response to infectious agents causes tissue damage and the release of sequestered antigens that activate autoreactive T cells (50, 51). Therefore, it is reasonable that infection can trigger disease flare-ups and lead to the increased administration of immunosuppressive medications, which can, in turn, contribute to disease damage. Our study reported that the most common infectious diseases were varicella zoster infection, followed by pneumonia and sepsis. Infections such as sepsis can also cause complications and further organ damage, compounding the impact on disease severity. Understanding these interactions is crucial for effectively managing SLE and preventing further organ damage associated with infections.

This study had some limitations. First, its retrospective study design meant that some data were missing; the SLEDAI-2K scores in each visit were estimated based on the available information in the medical records. Second, given the inability to track individuals into adulthood, we were unable to detect organ damage—particularly in the cardiovascular system—that might develop later in life. Third, some organ damage, such as spinal fractures, was detected based on clinical and physical examination. We did not perform imaging screening in all of the patients; in particular, those who did not have observable signs and symptoms were not screened. However, regular eye exams were routinely performed. To address these limitations and gain a more comprehensive understanding of disease progression, further studies with a prospective design are recommended.

## 5. Conclusion

One-third of patients with cSLE had permanent organ damage, representing an incident rate of 5.65 events per 100 person-years. The most common types of organ damage were ocular, musculoskeletal, and neurological. We identified high SLEDAI-2K scores at the time of initial presentation, MMF therapy, and infection as predictors of organ damage. Importantly, patients who have SLEDAI-2K scores ≥12 at disease diagnosis should be closely monitored for organ damage, especially during the 6.5-year period that represented the median time to disease damage in our study. Although managing the disease is crucial, physicians should also be aware of the potential adverse effects of medication, particularly corticosteroids. To minimize the risks associated with the prolonged use of high-dose corticosteroids, considering alternative immunosuppressive drugs for disease control may be beneficial. Additionally, routine monitoring for disease damage can help identify and address any signs of further irreversible organ impairment in patients with cSLE. Adopting these measures and being aware of the associated factors can prevent additional organ damage and enhance the overall quality of life in patients with cSLE.

## Data Availability

The original contributions presented in the study are included in the article/**Supplementary Material**, further inquiries can be directed to the corresponding author/s.
