# Supplementary material for: Risk factors associated with multiple organ damage in childhood-onset systemic lupus erythematosus
Source: Front Pediatr. 2023 Nov 28;11:1301201. doi: 10.3389/fped.2023.1301201 (PMC10717112; doi:10.3389/fped.2023.1301201)
Supplement: Supplementary file 1 [file Datasheet1.pdf]

Supplementary Material

Risk Factors Associated with Multiple Organ Damage in Childhood-Onset Systemic Lupus Erythematosus

Thanaporn Puengpipattrakul, Butsabong Lerkvaleekul, Kwanchai Pairojsakul, Soamarat Vilaiyuk*

*** Correspondence:** Soamarat Vilaiyuk: soamarat21@hotmail.com

1. Supplementary Figure and Tables

Figure S1. Patients' flow chart

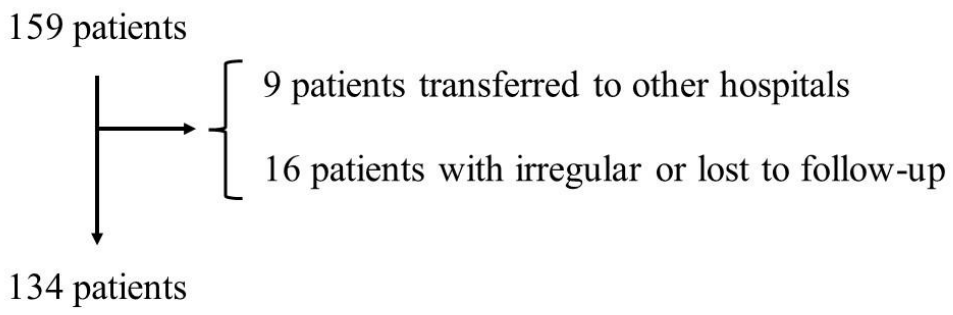

Table S1. Frequency of organ damage by domains.

**Disease damage by organ/system**	**Frequency (%)**
Ocular	15 (11.1)
- Cataract	11 (8.2)
- Retinal change or optic atrophy	4 (2.9)
Neuropsychiatric	10 (7.4)
- Cognitive impairment or major psychosis	5 (3.7)
- Seizures requiring therapy for 6 months	3 (2.2)
- Cerebrovascular accident ever	4 (2.9)
- Cranial or peripheral neuropathy	0 (0)
- Transverse myelitis	0 (0)
Renal	2 (1.5)
- Estimated or measured GFR < 50%	0 (0)
- Proteinuria	0 (0)
- End stage renal disease	2 (1.5)
Pulmonary	2 (1.5)
- Pulmonary hypertension	1 (0.7)
- Pulmonary fibrosis	0 (0)
- Shrinking lung	1 (0.7)
- Pleural fibrosis	0 (0)
- Pulmonary infarction	0 (0)
Cardiovascular	0 (0)
- Angina or coronary artery bypass	0 (0)
- Myocardial infarction ever	0 (0)
- Cardiomyopathy (ventricular dysfunction)	0 (0)
- Valvular disease	0 (0)
- Pericarditis for 6 months	0 (0)
Peripheral vascular	1 (0.7)
- Claudication for 6 months	0 (0)
- Minor tissue loss	0 (0)
- Significant tissue loss ever	0 (0)
- Venous thrombosis with swelling, ulceration or venous stasis	1 (0.7)
Gastrointestinal	0 (0)
- Infarction or resection of bowel, spleen, liver or gall bladder ever	0 (0)
- Mesenteric insufficiency	0 (0)
- Chronic peritonitis	0 (0)

- Stricture or upper gastrointestinal tract surgery ever<br>- Pancreatic insufficiency requiring enzyme replacement or with pseudocyst	0 (0)<br>0 (0)
Musculoskeletal	12 (8.9)
- Muscle atrophy or weakness<br>- Deforming or erosive arthritis<br>- Osteoporosis with fracture or vertebral collapse<br>- Avascular necrosis<br>- Osteomyelitis	0 (0)<br>0 (0)<br>6 (4.4)<br>7 (5.2))<br>0 (0)
Skin	2 (1.5)
- Scarring chronic alopecia<br>- Extensive scarring or panniculum other than scalp<br>- Skin ulceration for > 6 months	2 (1.5)<br>0 (0)<br>0 (0)
Premature gonadal failure	1 (0.7)
Diabetes	3 (2.2)
Malignancy	1 (0.7)

Table S2. Infectious agents in SLE patients (n = 74 events).

**Infectious diseases**	**Number (events)**	**Causes of infection**
- Chickenpox/Shingles	18	Varicella zoster
- Pneumonia	15	Unknown (n=6), Respiratory syncytial virus (n=2), Mycoplasma pneumoniae (n=1), Haemophilus influenzae (n=1), Rhinovirus (n=1), Cytomegalovirus (n=1), Aspergillus spp. (n=1), Pneumocystis jirovecii (n=1), Pseudomonas aeruginosa and Klebsiella pneumoniae (n=1)
- Sepsis	10	Unknown (n=5), Salmonella (n=2), Staphylococcus epidermidis (n=1), Streptococcus pneumoniae (n=1), Streptococcus bovis (n=1)
- Urinary tract infection	8	Escherichia coli (n=4), Klebsiella pneumoniae (n=1), Enterococcus faecalis (n=1), Streptococcus gallolyticus (n=1), Unknown (n=1)
- Bone and soft tissue infection	6	Unknown (n=4), Staphylococcus aureus (n=1), Salmonella (n=1)
- Gastroenteritis	4	Unknown (n=2), Salmonella (n=1), Norovirus (n=1)
- Central nervous system infection	4	Mycobacterium tuberculosis (n=1), dengue virus (n=1), Listeria monocytogenes,<br>Mycobacterium tuberculosis and herpes simplex virus (n=1), Unknown (n=1)
- Peritonitis	2	Candida spp. (n=1), Unknown (n=1)
- Pericarditis	1	Aspergillus spp. (n=1)
- Others	6	Cytomegalovirus (n=2), Influenza virus (n=1), Coronavirus (n=1), Dengue virus (n=1), Non-tuberculous mycobacteria (n=1)
